# Unilateral adrenal infarction in pregnancy with associated acute hypoadrenalism and subsequent spontaneous biochemical and radiological resolution

**DOI:** 10.1002/ccr3.5442

**Published:** 2022-02-11

**Authors:** Najeeb Shah, Harshal Deshmukh, Muhammad Jawaid Akbar, Yamna Saeed, Shahzad Akbar, Shah Malik, Belinda Allan

**Affiliations:** ^1^ Hull University Teaching Hospitals NHS Trust Hull Royal Infirmary Hull UK; ^2^ 4019 University of Hull Hull UK; ^3^ 8749 York and Scarborough Teaching Hospitals NHS Foundation Trust York UK

**Keywords:** hypoadrenalism in pregnancy, hypotension in pregnancy, medical problems in pregnancy, unilateral adrenal infarction

## Abstract

Adrenal infarction is a rare cause of abdominal pain during pregnancy, and if missed, it can result in devastating clinical consequences for the mother and the child. The authors report a case of a young female who presented with severe abdominal pain and nausea. The biochemistry showed raised inflammatory markers and significant lactic acidosis. As the cause of the symptoms was not clear and the patient continued to deteriorate, a contrast‐enhanced CT abdomen and pelvis was done which was suggestive of an acute left adrenal infarction. Subsequently, the patient was confirmed to have biochemical hypoadrenalism and required replacement doses of hydrocortisone until recovery of the adrenal glucocorticoid reserve and anticoagulation for the duration of pregnancy. We discuss the workup including diagnostic imaging, follow‐up, and considerations for future pregnancies in this case.

## Graphical abstract

Pregnancy predisposes to Adrenal infarction (AI) as it is a hypercoagulable state, however, its occurrence remains rare. Abdominal pain is a frequent symptom but delineating its cause during gestation is challenging, and therefore, clinicians must suspect AI especially when the symptoms are sudden, severe, and associated with nausea/vomiting or fever.

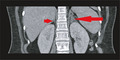

## 1 INTRODUCTION

Non‐hemorrhagic adrenal infarction (NHAI) is a rare cause of acute abdominal pain during pregnancy which often presents in the third trimester.^1^ There is a paucity of clinical reports describing this clinical entity, with <20 such cases published in literature so far.^1^, ^2^, ^3^, ^4^, ^5^, ^6^, ^7^, ^8^, ^9^ The location of the pain varies from case to case and may occur in the flanks, abdomen, or chest. Recognition of adrenal infarction as a cause of abdominal pain during gestation is challenging due to the obscurity of this diagnosis and the fact that during evaluation of such cases, attention usually turns to the usual culprits, for example, physiological (uterine enlargement/increase volume of amniotic fluid), preterm labor, placental abruption, appendicitis, cholecystitis, and pulmonary embolism. Once suspected, the diagnosis of NHAI presents a challenge as imaging options in pregnancy are limited by diagnostic accuracy and risk of fetal radiation exposure. Furthermore, hypoadrenalism is not expected in unilateral NHAI. We describe a case of a young female in the third trimester of pregnancy who presented with left flank pain and after the initial investigations returned normal, a contrast‐enhanced computed tomography (CT) scan of the abdomen and pelvis revealed left adrenal infarction. Subsequently, the morning (0900) serum cortisol was found to be grossly abnormal at 78 nmol/L (200–700). The patient was commenced on hydrocortisone therapy and anticoagulated with warfarin.

This case is unique as not only does it describe a rare diagnosis, it highlights that unilateral adrenal infarction can lead to adrenal insufficiency and hemodynamic instability.

## 2 CASE PRESENTATION

A 25‐year‐old female, with her first pregnancy and 32 weeks into gestation, presented to the antenatal day unit with acute onset left flank pain. Initially thought to be musculoskeletal pain, the patient was discharged on simple analgesia. Later, the same day, in addition to the left flank pain, she developed sharp, central lower chest pain, back pain and associated vomiting. Pain score was 9/10. A few hours later, the patient developed right‐sided abdominal pain. There was no associated fever or diarrhea. There was no history of trauma. There was no preceding illness to report, and up to this point, the pregnancy had been progressing as per normal with a normal fetal anomaly scan a few months earlier. There was no past medical or surgical history of note nor was she taking any regular medication. There was no personal or family history of thromboembolic disease. She was employed as an assistant manager of a shop which did not require strenuous physical activity.

On clinical examination her initial blood pressure (BP) was 112/58 which later dropped to 100/64 with the heart rate (HR) 101108 beats/min. Her oxygen saturations were 100% on room air with a respiratory rate of 24–26 breaths/min. There was evidence of generalized mild abdominal tenderness.

She was noted to have a lactic acidosis of 9.0 mmol/L (0.5–1.0) with a bicarbonate of 13.8 mmol/L (23–28) and glucose of 10 mmol/L on a venous blood gas (VBG) analysis. The electrocardiogram (ECG) did not show acute ischemia or right heart strain pattern while the chest X‐ray excluded pneumonia and air under diaphragm. Urine dip stick results did not suggest infection or significant protein loss but did show glucose and ketones 3+ with blood 2+. Mid‐stream urine culture was negative for organisms, and an ultrasound of the abdomen was unremarkable. She was reviewed by the obstetrics team who thought this may be spontaneous rupture of membranes with septicemia while the surgical team felt this could be a case of an acute abdomen, that is, appendicitis/pancreatitis. Her initial blood test results (summarized in the Table 1) and the clinical picture pointed to a diagnosis of intra‐abdominal septicemia in view of raised inflammatory markers, lactic acidosis, and low bicarbonate levels. She was commenced on broad‐spectrum intravenous antibiotics, intravenous fluids, and analgesia.

**TABLE 1:** Initial blood test results

Blood test	Result	Normal
Sodium	**134 mmol/L**	135–144
Potassium	4.1 mmol/L	3.5–5.3
Urea	2.5 mmol/L	3–7.6
Creatinine	48 umol/L	55–87
CRP	**38 mg/L**	0–8
PT	11.4 s	10–13.5
APTT	25.9 s	28–38
Hemoglobin	115 g/L	120–160
WCC	**23.3 × 10^9^/L**	4–11
Platelets	446 × 10^9^/L	150–400
MCV	80.9 fL	80–100
D dimer	**673 ng/mL**	0–300
Amylase	**32 U/L**	33–153
PH	**7.28**	7.35–7.45
Bicarbonate	**13.8 mmol/L**	23–26
Base excess	**−13.3 mmol/L**	+2 to −2
Lactate	**9.0 mmol/L**	0–1

Abbreviations: APTT, activated partial thromboplastin time; CRP, C‐reactive protein; MCV, mean corpuscular volume; PT, prothrombin time; WCC, white cell count.

The values appearing in bold depict an abnormal result.

**FIGURE 1:**
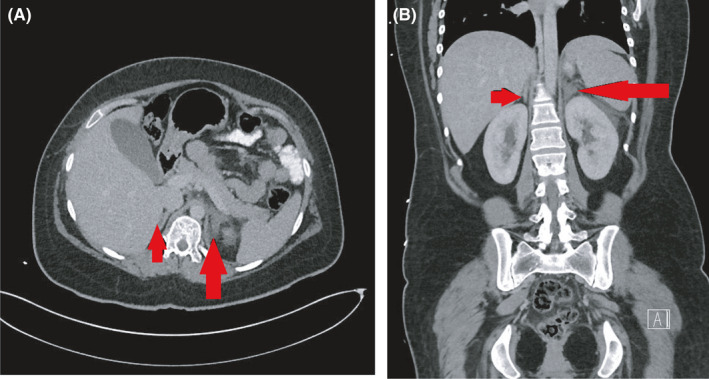
(A) Contrast‐enhanced CT abdomen and pelvis in the axial view. The arrow shows an enlarged left adrenal gland with low attenuation when compared with the right. (B) Contrast‐enhanced CT abdomen and pelvis in the coronal view. The arrow depicts an enlarged left adrenal gland with low attenuation when compared with the right

In view of unexplained severe symptoms, raised inflammatory markers, and significant lactic acidosis, a contrast‐enhanced CT scan of the abdomen and pelvis was done after discussing benefits and risks with the patient. This revealed an enlarged left adrenal gland with low attenuation in keeping with infarction (Figure 1A,B).

**FIGURE 2:**
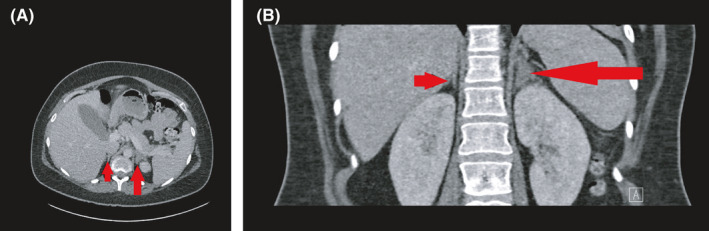
(A) Contrast‐enhanced CT adrenal gland in the axial view. The arrow clearly shows an enlarged and edematous left adrenal gland compared with the right. (B) Contrast‐enhanced CT adrenal gland in the coronal view. An enlarged and edematous left adrenal gland can clearly be visualized compared with the right

The following day random serum cortisol was done at 1410 h which came back at 557 nmol/L (200–700). The patient had spontaneous rupture of membranes, and a female baby weighing 2.2 lbs was delivered by an emergency C‐section who then required admission to the neonatal intensive care unit for breathing difficulties, however, thankfully, did go on to make a full recovery. The mother continued to have abdominal and back pain and became hypotensive. Metabolic and lactic acidosis improved following conservative management. On Day 3, postpartum (0900 h) serum cortisol was 78 nmol/L (200–700). Bilateral adrenal infarction was suspected, and a dedicated contrast‐enhanced adrenal CT scan was done which confirmed left adrenal infarction with low attenuation and edema but the right adrenal gland was radiologically normal (Figure 2A,B).

As at the time she was clinically well and hemodynamically stable, a parenteral dose of hydrocortisone was not required and instead, she was commenced on hydrocortisone 20 mg at 0800 h and 10 mg at 1400 h and anticoagulated with warfarin to prevent progression of the infarction and contralateral thrombosis. Once her symptoms resolved, she was discharged with endocrinology follow‐up. Four months after the initial event, she was reviewed in the endocrinology clinic with a short synacthen test (SST) which revealed T^0^ cortisol 263 nmol/L and T^30^ 407 nmol/L (synacthen 250 mcg IM was administered after taking a blood sample for T^0^ cortisol. A peak cortisol of ≥540 nmol/L was regarded as a normal short synacthen test).

**FIGURE 3:**
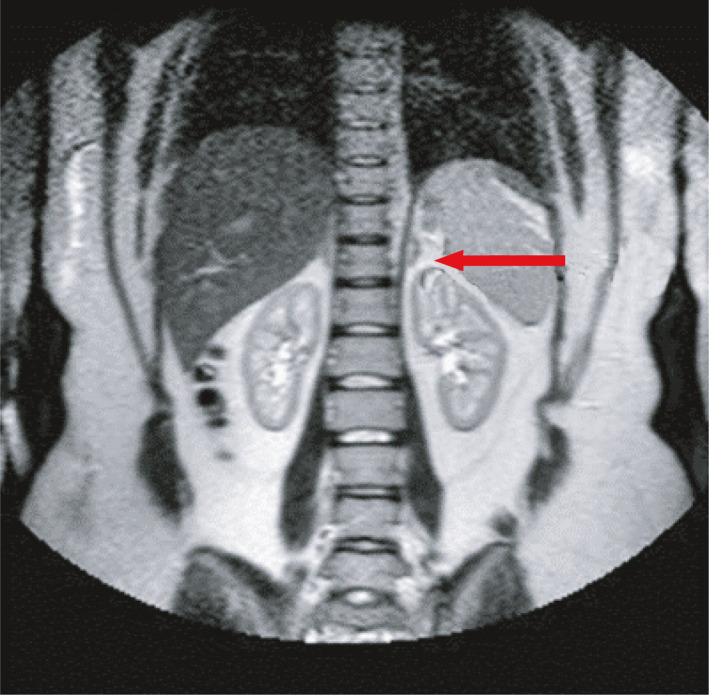
MRI abdomen in the axial view showing a normal left adrenal gland

She was maintained on hydrocortisone 20 mg at 0800 h and 10 mg at 1400 h while her anticoagulation in the form of warfarin was discontinued. Two months later, she was seen with an MRI of her abdomen, which showed complete resolution of the left adrenal infarction (Figure 3).

The dose of hydrocortisone was reduced to 10 mg at 0800 h and 5 mg at 1400 h. Three months later, she was reviewed again with a repeat SST which showed T^0^ cortisol 150 nmol/L and T^30^ cortisol 585 nmol/L. She was maintained on hydrocortisone 10 mg at 0800 h and 5 mg at 1400 h. Seven months later her steroid reserve had completely recovered with SST showing T^0^ cortisol 529 nmol/l and T^30^ cortisol 661 nmol/L (Table 2 summarizes serial SST results). Hydrocortisone at this visit was discontinued, and the patient was discharged from follow‐up. Apart from pregnancy, no other thrombogenic cause was found. Prophylactic anticoagulation with low molecular weight heparin (LMWH) was recommended for the duration of any future pregnancies.

**TABLE 2:** Serial SST results

Time	SST – Cortisol (nmol/L)		Hydrocortisone dose (mg)
4 months post event	T^0^ 263	T^30^ 407	20/10
9 months post event	T^0^ 150	T^30^ 585	10/5
16 months post event	T^0^ 529	T^30^ 661	Discontinued

Note

Synacthen 250 mcg IM was administered after taking a blood sample for T^0^ cortisol. A peak cortisol of ≥540 nmol/L was regarded as a normal short Synacthen test.

## 3 DISCUSSION

Adrenal infarction has been reported with hypercoagulable states including antiphospholipid syndrome,^10^ myelodysplasia,^11^ Crohn's disease,^12^ and more recently, COVID‐19 infection^13^ among other pathologies.

We present a case of unilateral adrenal infarction in pregnancy with a spontaneous biochemical and radiological resolution. Unilateral adrenal infarction in pregnancy is rare^3^, ^4^, ^14^ with a reported incidence of 1.3% on MRI scans at one institution done for abdominal pain.^15^

There are several predisposing factors for developing adrenal infarction in pregnancy. Pregnancy is a hypercoagulable state, which, from an evolutionary perspective, is a protective mechanism against bleeding due to miscarriage or during childbirth.^16^ Studies have shown that pregnant women are at a 4–5 fold increased risk of thromboembolism during pregnancy and the postpartum period compared with the non‐pregnant state.^16^ There are several reasons for this. Normal pregnancy is accompanied by increased concentration of factors VII, VIII, X, and von Willebrand factor and by pronounced increases in fibrinogen.^17^ Free protein S, the active, unbound form, is decreased during pregnancy secondary to increased levels of its binding protein, the complement component C4b.^17^ All these increase the likelihood of non‐hemorrhagic infarctions. This increased thrombogenic state is likely to affect the adrenal gland because of its unique vasculature with rich arterial supply but poor venous drainage.

Evaluation of acute abdominal pain in pregnancy is challenging because of an enlarged uterus, presence of varying volumes of amniotic fluid and change in the anatomy associated with pregnancy. The common non‐obstetric differential diagnosis for acute abdominal pain in pregnancy^18^, ^19^ that can be recognized with imaging are acute appendicitis, cholelithiasis, cholecystitis, pancreatitis, and bowel disease. However, adrenal infarction should be included in the differential diagnosis of severe upper abdominal pain in pregnancy,^3^, ^4^, ^14^ especially if there is a persistent abdominal and flank pain, and the above‐mentioned common causes are ruled out. The diagnosis can be made by an adrenal CT, contrast‐enhanced MRI, or a non‐contrast MRI scan. However, adrenal CT and contrast‐enhanced MRI are generally avoided during pregnancy due to fetal radiation exposure,^20^ and non‐contrast MRI is the investigation of choice. In a recent literature review, interestingly, Chagué et al. report that among 17 cases of NHAI, CT scan was done in 13 cases while an MRI was done on 9 instances.^1^ This may be because an urgent CT scan is more readily available and reliably yields diagnostic information.

There is no consensus about the sequence of SST and imaging in these situations. However, an SST can be done prior to imaging in high‐risk patients with personal or family history of autoimmune disease, malignancy, or in patients who refuse abdominal scanning. In our patient, the SST clearly demonstrated adrenal insufficiency, and she was started on steroid replacement. Interestingly, the right adrenal gland was radiologically completely normal on the CT scan; however, it did not compensate for the adrenal insufficiency. It is possible that the changes in the right adrenal gland were subtle and not visualized on the initial CT scan.

There is no consensus on the follow‐up imaging and reassessment of adrenal reserves in this group of patients. In our patient, we repeated an MRI scan and SST approximately 6 months after the first scan. Interestingly, her MRI scan at follow‐up showed complete resolution of the infarction, and the SST confirmed biochemical resolution of the adrenal insufficiency. There are very few reports in the literature documenting the spontaneous resolution of pregnancy‐related adrenal infarcts, and our report highlights the importance of repeat scanning and repeat SST in this group of patients.

In the setting of adrenal infarction, the patient was anticoagulated to prevent contralateral thrombosis. Warfarin was chosen to anticoagulate the patient as she had already delivered her child. Use of coumarin derivatives by the mother at any time during pregnancy may increase the risk of central nervous system anomalies in the fetus.^21^ There is also no consensus on the duration of anticoagulation in such cases at present. It is reasonable to consider unilateral adrenal infarction in pregnancy as a provoked venous thromboembolism event in the absence of thrombogenic disease and as such 3–6 months of anticoagulation is a reasonable approach.

This case highlights that it may take up to 6 months before restoration of the adrenocortical steroid reserve, and thus, patients should be followed up with SST’s under endocrinology for this time period at least. Once unilateral NHAI is confirmed, patients should be offered anticoagulation to prevent progression of infarction and contralateral thrombosis. Benefits vs. risks of anticoagulation should always be considered, and where possible, patients should always be included in the decision‐making process. More studies are required to ascertain a safe and effective duration of anticoagulation in such cases. In patients who develop adrenal infarction during gestation; we recommend prophylactic anticoagulation for the duration of subsequent pregnancies. After the acute event, follow‐up adrenal imaging should be considered as patients may develop adrenal hemorrhage due to revascularization or atrophy of the gland. Follow‐up imaging will also help to assess recovery of the adrenal gland, which may take up to 6 months, as described by our case.

## 4 CONCLUSION

Increased concentration of endogenous procoagulants and venous stasis increases the risk of adrenal infarction in pregnancy. This case asserts the importance of suspecting adrenal infarction in pregnant women presenting with abdominal pain, nausea/vomiting, and hemodynamic instability.

## CONFLICT OF INTEREST

There are no conflicts of interest to declare from any author with regards to this publication.

## AUTHOR CONTRIBUTION

Najeeb Shah, the first and corresponding author, conceptualized the project, obtained informed written consent, wrote the first draft, and did all subsequent revisions of the manuscript. Harshal Deshmukh assisted with the drafting of the manuscript, undertook the literature search, and made constructive comments to improve the quality of the manuscript. MJ Akbar obtained all the suitable radiological images and made comments to improve the quality of the manuscript. Yamna Saeed, Shah Malik, and Shahzad Akbar made comments to improve the quality of the manuscript and edited the final draft. Belinda Allan conceptualized the project, made comments to improve the quality of the manuscript, and provided overall supervision of the project as the senior author.

## CONSENT

Written informed consent was obtained from the patient for the publication of this case report and associated images.

## Data Availability

As this is a case report, anonymized data can be made available by the authors upon reasonable request subject to local confidentiality and ethics guidelines.
